# Huangqi Guizhi Wuwu Decoction can prevent and treat oxaliplatin-induced neuropathic pain by TNFα/IL-1β/IL-6/MAPK/NF-kB pathway

**DOI:** 10.18632/aging.203794

**Published:** 2022-06-27

**Authors:** Mingzhu Li, Zheng Li, Xiande Ma, Shengbo Jin, Yang Cao, Xuebing Wang, Jian Zhao, Jianbo Wang, Xin Wang, Jian Xu

**Affiliations:** 1 Department of Integrated Traditional Chinese and Western Medicine Medical Oncology, Cancer Hospital of China Medical University, Liaoning Cancer Hospital and Institute, Shenyang 110042, Liaoning Province, P.R. China; 2 Liaoning University of Traditional Chinese Medicine, Shenyang 110085, Liaoning Province, P.R. China; 3 The First Affiliated Hospital of Liaoning University of Traditional Chinese Medicine, Shenyang 110085, Liaoning Province, P.R. China; 4 Department of Gynecology Cancer Hospital of China Medical University, Liaoning Cancer Hospital and Institute, Shenyang 110042, Liaoning Province, P.R. China; 5 Department of Colorectal Surgery, Cancer Hospital of China Medical University, Liaoning Cancer Hospital and Institute, Shenyang 110042, Liaoning Province, P.R. China; 6 Department of Hepatobiliary Surgery, Cancer Hospital of China Medical University, Liaoning Cancer Hospital and Institute, Shenyang 110042, Liaoning Province, P.R. China

**Keywords:** Huangqi Guizhi Wuwu Decoction, oxaliplatin, CINP, MAPK/NF-kB pathway

## Abstract

Objective: This study explored the effects and mechanisms of Huangqi Guizhi Wuwu Decoction on chemotherapy-induced neuropathic pain (CINP).

Methods: Bodyweight and related behavioral testing of the rat model were utilized to investigate the effects of Huangqi Guizhi Wuwu Decoction on CINP. ELISA was used to measure the levels of TNF-α, IL-1β, and IL-6, in the serum of chronic CINP rats. Immunohistochemistry and Western blot analysis were performed to detect the expression of MAPK pathway related-proteins namely ERK1/2, p38, and JNK, and the expression of downstream essential proteins such as c-Fos, CREB, and NF-κB.

Results: Body weight and related behavioral testing of the rat model suggests that Huangqi Guizhi Wuwu Decoction can improve the slow weight gain of oxaliplatin-induced chronic CINP model rats and effectively prevent and treat oxaliplatin-induced regular CIPN rat model of hyperalgesia. It can also oppress the mechanical pain threshold, cold pain threshold, and heat pain threshold decreased. Furthermore, by ELISA, immunohistochemistry, and western blot analysis, we found that Huangqi Guizhi Wuwu Decoction can down-regulate the levels of TNF-α, IL-1β, and IL-6 in the serum of chronic CINP rats induced by oxaliplatin. It also suppresses the expression of MAPK pathway related-proteins ERK1/2, p38, and JNK. This results in a decrease in the expression of downstream essential proteins, c-Fos, CREB, and Nf-κB.

Conclusions: In conclusion, we found that Huangqi Guizhi Wuwu Decoction can combat nerve cell injury, reduce pain sensitization, and prevent and repair the damage of nerve cells in the oxaliplatin CINP model rats via TNFα/IL-1β/IL-6/MAPK/NF-kB pathway.

## INTRODUCTION

In the past few years, the incidence and mortality rate of malignant tumors in China have been on the rise, posing significant threats to public health [1]. Pain treatment has always been an essential part of cancer treatment since it plays an important role in the prevention and treatment of common adverse effects of cancer therapy, especially chemotherapy-induced neuropathic pain (CINP). CINP is a kind of intractable pain [2], and its prevalence varies from drug to drug, ranging from 19% to 85% [3]. Oxaliplatin is a first-line chemotherapy drug used to treat gastrointestinal cancer. Importantly, the incidence of oxaliplatin-induced CINP is reported to be between 76%-90% [4–6]. Its clinical manifestations included a rapid onset of neuropathy aggravated by cold, which usually develops into chronic neuropathy after several treatment cycles [7, 8]. It hinders the continuity of tumor treatment and disease control rate and seriously affects the quality of life of the patients. Thus, oxaliplatin CINP, is an urgent clinical problem to be solved.

Huangqi Guizhi Wuwu Decoction was first described in a traditional Chinese medicine book, *Jin Kui Yao Lue*. It was suggested to be used for *Xue Bi*, a term capturing the clinical manifestations of neuropathic pain, such as pain, numbness sensory disturbance, cold limbs, and aggravation of cold [9–11]. The meta-analysis of Huangqi Guizhi Wuwu Decoction shows that it has a better clinical effect in preventing oxaliplatin-induced peripheral nerve injury compared with conventional western medicine [12–15]. However, how Huangqi Guizhi Wuwu Decoction affects CINP has not been fully understood.

Previous studies have found that both the mechanism of oxaliplatin-induced chronic neuropathic pain and Western medicine treatment mechanism have possible associations with the MAPK signaling pathway [16–18]. Furthermore, a cluster analysis of network pharmacology of Huangqi Guizhi Wuwu Decoction also indicated that the MAPK signaling pathway might play a role in the treatment of neuropathic pain [13]. Therefore, in this study, we established a rat model of chronic oxaliplatin CINP and compared the effects of oral and topical use of Huangqi Guizhi Wuwu Decoction on hyperalgesia and neurocyte injury in rats. Our findings suggest that Huangqi Guizhi Wuwu decoction can prevent and repair CINP induced by oxaliplatin by regulating the MAPK signaling pathway. The mechanism study of nerve cell injury in rats can provide a theoretical basis for the prevention and treatment of oxaliplatin-induced CINP, lay a scientific and favorable objective basis for clinical application, and promote the development and application of traditional Chinese medicine.

## MATERIALS AND METHODS

### Rat model grouping and building

Forty-five female Sprague-Dawley (SD) rats were randomly divided into five groups. Normal control group (A group), model group (oxaliplatin chemotherapy-induced chronic neuropathic pain model, B group), Western prevention and treatment group (oxaliplatin-induced pain model + duloxetine gavage, C group), Traditional Chinese medicine gavage prevention and treatment group (oxaliplatin-induced pain model + Huangqi Guizhi Wuwu Decoction gavage, D group), and Traditional Chinese medicine is socking prevention and treatment group (oxaliplatin-induced pain model + Huangqi Guizhi Wuwu Decoction socking, E group).

Xiao et al. built the model of oxaliplatin chemotherapy-induced neuropathic pain [19]. In this study, the dosage of oxaliplatin was 2 mg/kg/d. On days 1, 2, 3, 4, and 5, oxaliplatin was intraperitoneally injected to construct a chronic neuropathic pain model. When the rats showed hyperalgesia symptoms such as unwillingness to move forward, lifting, licking, even retreating, mechanical pain threshold, as well as cold and hot pain threshold decreased. According to these results, the model was considered to be successful.

We followed the guidelines of J*in Kui Yao Lue* to formulate our Huangqi Guizhi Wuwu Decoction. It was composed of Huangqi (9g), Guizhi (9g), Peony (9g), Ginger (18g), Jujube (10g). These were added to water, and the mixture was brought to a boil.

The Chinese medicine soaking, and the immersing method was conducted as follows: After depilating the limbs of the rats, we fixed the rats with a bubble immersing device. The knee joints, elbow joints, and tails of the rats were soaked in 35° C Chinese medicine. The mouths of the rats were exposed to prevent them from choking water and licking drugs. After being washed, the rats were immersed in warm water, and their fur was dried.

### Bodyweight and related behavioral testing

According to a previous study, bodyweight, mechanical withdrawal threshold, cold pain threshold, and thermal pain threshold were evaluated [20].

### Elisa

Serums samples were obtained from rats, and the levels of TNFα (SCA133Ra), IL-1β (SEA563Ra), and IL-6 (SEA079Ra) were detected by the Elisa kit following the protocols of the manufacturer.

### Immunohistochemistry (IHC) analysis

L4-L5 dorsal root ganglions of the spinal cords of the rats were obtained and fixed in 4% formaldehyde. Then these dorsal root ganglions were embedded in paraffin. Subsequently, the paraffin specimens were sliced into 6-μm sections, deparaffinized by xylene and rehydrated through a graded ethanol series.

Following that, the sections were stained with hematoxylin and eosin to investigate the changes of pathological morphology of L4-L5 spinal dorsal root.

Then, the specimens were incubated with a primary antibody against ERK1/2 (1:50; Abcam, ab54230), p38 (1:250; Abcam, ab170099), JNK (1:1000; Abcam, ab208035), C-Fos (1:800, Abcam, ab208942) CREB (1:400; Abcam, ab32515), and NF-kB (1:200; Abcam, ab16502) at 4° C overnight to explore the expressions of ERK1/2, p38, JNK, C-Fos, CREB, and NF-kB. Afterwards, they were incubated with a secondary antibody (Gene Tech Co. Ltd., Shanghai, China) for 60 min and with a DAB kit (Gene Tech Co. Ltd.) for 10 min. The evaluation of ERK1/2, p38, and JNK expression was conducted according to previous studies [21].

### Western blotting

L4-L5 dorsal root ganglions of spinal cords of the rats were obtained and then lysed in RIPA lysis buffer. The tissue lysates (40 μg/lane) were separated via 10% SDS-polyacrylamide gel electrophoresis and were transferred to PVDF membranes. After being blocked by 5% fat-free dry milk in TBST for 1 hour, the membranes were incubated with anti-human ERK1/2 antibody (1:300; Abcam, ab17942), anti-human p38 antibody (1:2000; Abcam, ab170099), anti-human JNK antibody (1:2000; Abcam, ab208035), anti-human C-Fos antibody (1:2000, Abcam, ab190289), anti-human CREB antibody (1:2000, Abcam, ab32515), anti-human NF-kB antibody (1:500, Abcam, ab19870), and anti-human GAPDH antibody (1:3000, Abcam, ab8245) overnight at 4° C. On the second day, the PVDF membranes were incubated with a goat anti-rabbit IgG (Zhong Shan Jin Qiao Co. Ltd.) for one hour after immersed with TBS three times, and immunoreactive bands were visualized by an enhanced chemiluminescent reagent.

### Statistical analysis

SPSS 22.0 software was used for statistical analysis. All data were expressed as mean ± standard deviation (x ± s) and were analyzed by independent sample t-tests. *P*< 0.05 was considered statistically significant.

## RESULTS

### The effect of Huangqi Guizhi Wuwu Decoction on body weight of oxaliplatin CINP model rats

**Figure 1:**
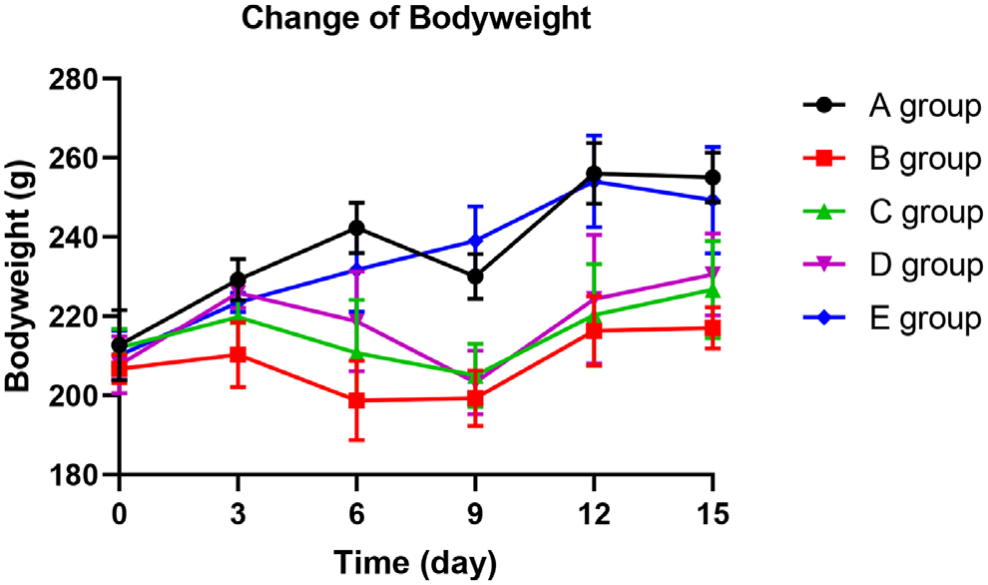
The effect of Huangqi Guizhi Wuwu Decoction on body weight of different rat groups.

With the application of oxaliplatin, groups B, C, and D exhibited a significantly slower weight gain from day 6 compared with group A (*P*=0.014, *P*=0.026, and *P*=0.034, respectively). In group D, the weight gain slowed down until the 12^th^ day. In groups B and C, the weight gain slowed down until the 15^th^ day. However, there was no significant difference in weight gain among groups A and E (*P*=0.254). These finding show that Huangqi Guizhi Wuwu Decoction might prevent slow weight gain (see Figure 1).

### The effect of Huangqi Guizhi Wuwu Decoction on mechanical withdrawal threshold of oxaliplatin CINP model rats

With the application of oxaliplatin, the mechanical withdrawal threshold of group B decreased gradually from the 9^th^ day until the 15^th^ day. The mechanical withdrawal threshold of group B was significantly lower than group A on the 9^th^ day (*P*=0.005), 12^th^ day (*P*=0.003), and 15^th^ day (*P*<0.001).

**Figure 2:**
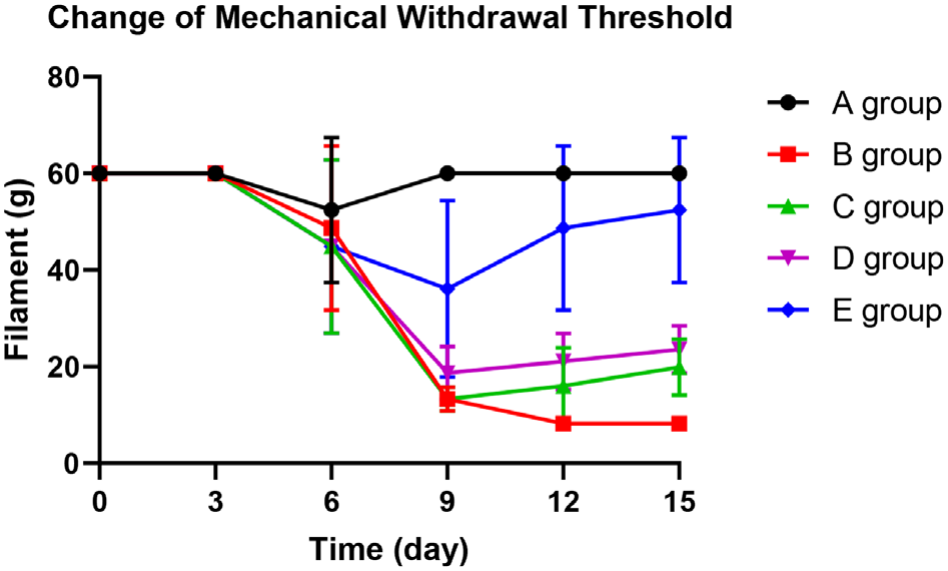
The effect of Huangqi Guizhi Wuwu Decoction on mechanical withdrawal threshold of different rat groups.

On the 12^th^ day and 15^th^ days, groups C and D could significantly prevent the decrease of mechanical withdrawal threshold compared with group B (*P*=0.023, *P*=0.015; *P*=0.031, *P*=0.024, respectively). However, group E could control the reduction of the mechanical withdrawal threshold more effectively. There was no decrease on the 3^rd^ day (*P*=0.425), 6^th^ day (*P*=0.342), 12^th^ day (*P*=0.127), and 15^th^ day (*P*=0.368) compared with group A. The results are shown in Figure 2.

### The effect of Huangqi Guizhi Wuwu Decoction on the cold pain threshold of oxaliplatin CINP model rats

**Figure 3:**
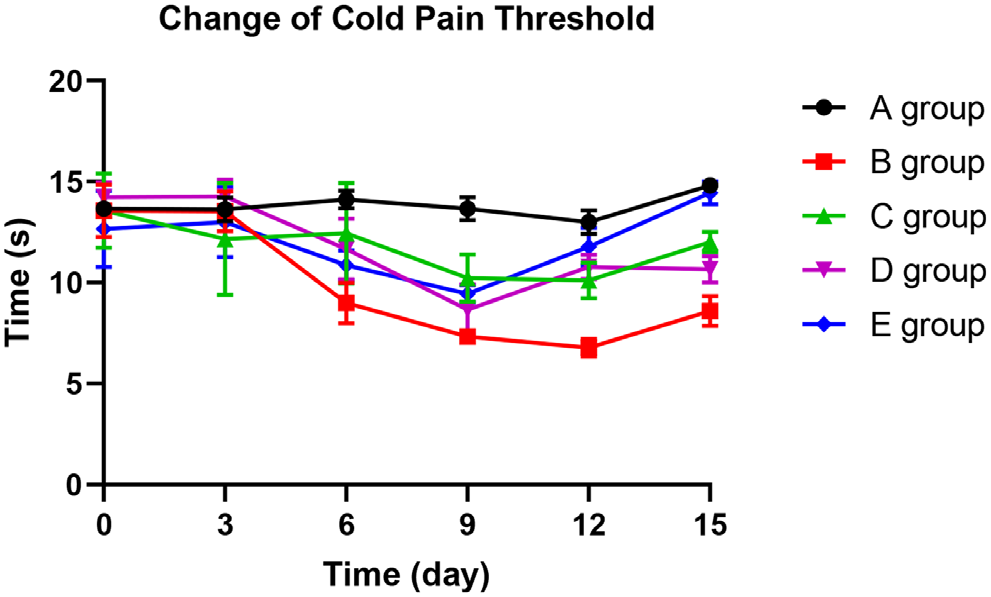
The effect of Huangqi Guizhi Wuwu Decoction on the cold pain threshold of different rat groups.

With the application of oxaliplatin, cold pain threshold of group B decreased gradually until the 15^th^ day. The mean pain threshold of group B was significantly lower than group A on the 6^th^ day (*P*=0.026), 9^th^ day (*P*=0.015), 12^th^ day (*P*=0.006), and 15^th^ day (*P*=0.023). Compared with group B rats, groups C and D could significantly prevent the decrease of cold pain threshold on the 6^th^ day, 12^th^ day, and 15^th^ day (*P*=0.012, *P*=0.026, *P*=0.014; *P*=0.013, *P*=0.019, *P*=0.021, respectively). Group E could control the reduction of cold pain threshold more effectively. No decrease was observed on the 3^rd^ day (*P*=0.658), 6^th^ day (*P*=0.057), and 15^th^ day (*P*=0.089) compared with group A. Group E was more effective than group C on the 12^th^ day and 15^th^ day (*P*=0.021; *P*=0.045, respectively). The results are displayed in Figure 3.

### The effect of Huangqi Guizhi Wuwu Decoction on the thermal pain threshold of oxaliplatin CINP model rats

**Figure 4:**
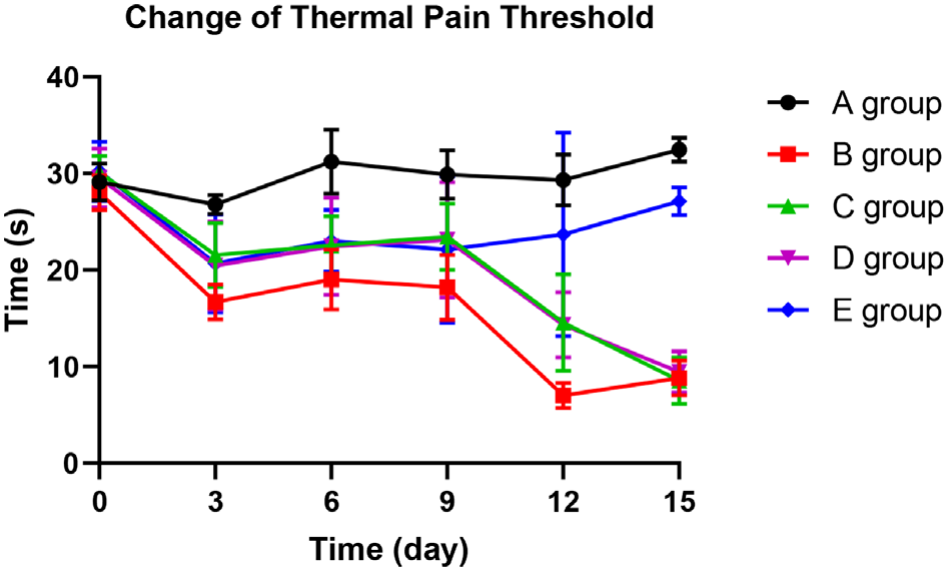
The effect of Huangqi Guizhi Wuwu Decoction on the thermal pain threshold of different rat groups.

With the application of oxaliplatin, the thermal pain threshold of group B decreased gradually until the 15^th^ day. The thermal pain threshold of group B was significantly lower than group A on the 3^rd^ day (*P*=0.015), 6^th^ day (*P*=0.024), 9^th^ day (*P*=0.023), 12^th^ day (*P*<0.001), and 15^th^ day (*P*<0.001). Compared with group B, group C could significantly prevent the decrease of thermal pain threshold on the 3rd and 9th days (*P*=0.035, *P*=0.031, respectively). On the 12^th^ day, group D could dramatically avoid the reduction of the thermal pain threshold (*P*=0.019) compared with group B. Group E could control the decrease in the thermal pain threshold more effectively. Group E showed no more decrease on the 9^th^ day, 12^th^ day, and 15^th^ day than group A (*P*=0.124, *P*=0.354, *P*=0.423). The results are exhibited in Figure 4.

### The effect of Huangqi Guizhi Wuwu Decoction on the morphology of nerve cells of L4-L5 dorsal root ganglions in oxaliplatin CINP model rats

**Figure 5:**
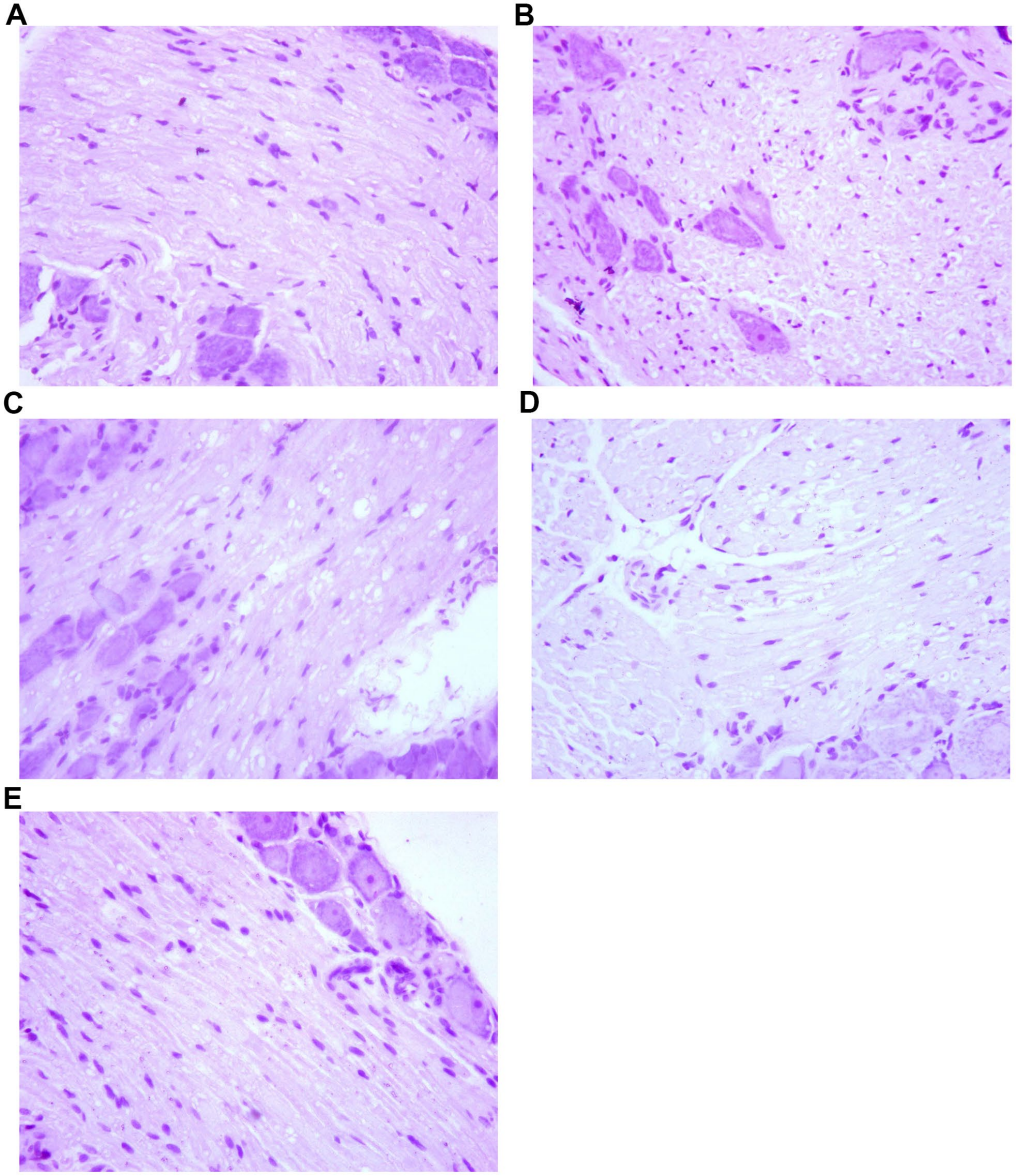
**The effect of Huangqi Guizhi Wuwu Decoction on the morphology of nerve cells of L4-L5 dorsal root ganglions of different rat groups.** (**A**–**E**) A group- E group.

By HE staining, we observed that the dorsal root ganglion cells of the spinal cord were arranged with regular cell spacing and standard nucleus size in group A. In contrast, the dorsal root ganglion cells were arranged disorderly in group B. The distance between cells was larger and the nucleus were smaller than usual, and the apoptotic cells could be seen, which was quite different from the normal nerve cells. In group C rats, the arrangement of dorsal root ganglion cells was generally regular, with larger cell spacing and slightly smaller nuclei than usual. The dorsal root ganglion cells in group D were arranged regularly. The cell spacing was bigger and the nucleus was somewhat smaller than usual, and a small number of cell apoptosis were observable. There was no significant difference between group C. However, in group E, the dorsal root ganglion cells were arranged regularly with normal cell spacing and normal nuclear size. There was no significant difference compared with normal nerve cells. The results are presented in Figure 5.

### The effect of Huangqi Guizhi Wuwu Decoction on the level of TNFα, IL-1β, and IL-6 in the serum of oxaliplatin CINP model rats

Compared with group A, the level of TNFα was higher in groups B-E. The difference was significant (*P*<0.001, *P*=0.012, *P*=0.021, and *P*=0.024, respectively). The level of TNFα was lower in group C-E rats compared with group B. The difference was significant (*P*=0.023, *P*=0.042, *P*=0.019). Compared with group C, there was no significant difference in the level of TNFα in group D and E. However, compared with group D, the level of TNFα was lower in group E. The difference was significant (*P*=0.047).

Compared with group A, the level of IL-1β was higher in groups B-E. The difference was significant (*P*<0.001, *P*=0.013, *P*=0.022, and *P*=0.025, respectively). Compared with group B, the level of IL-1β was lower in groups C-E. The difference was significant (*P*=0.021, *P*=0.038, *P*=0.018). Compared with group C, there was no significant difference in groups D and E for the level of IL-1β. However, compared with group D, the level of IL-1β was lower in group E. The difference was significant (*P*=0.046).

**Figure 6:**
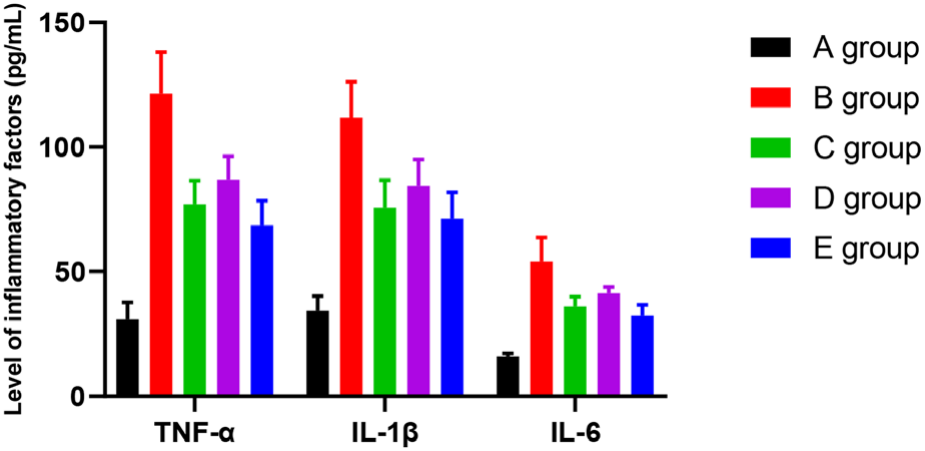
The effect of Huangqi Guizhi Wuwu Decoction on the level of TNFα, IL-1β, and IL-6 in the serum of different rat groups.

Compared with group A, the level of IL-6 was higher in group B-E. The difference was significant (*P*<0.001, *P*=0.003, *P*=0.012, and *P*=0.018, respectively). Compared with group B, the level of IL-6 was lower in groups C-E. The difference was significant (*P*=0.019, *P*=0.032, *P*=0.017). Compared with group C, the level of IL-6 was higher in group D (*P*=0.049), and there was no significant difference in group E (*P*=0.126). However, compared with group D, the level of IL-6 was lower in group E. The difference was significant (*P*=0.040). The results are given in Figure 6.

### The effect of Huangqi Guizhi Wuwu Decoction on the expression of ERK1/2, p38, JNK, c-Fos, CREB, and NF-kB in the L4-L5 dorsal root ganglions of oxaliplatin CINP model rats

**Figure 7:**
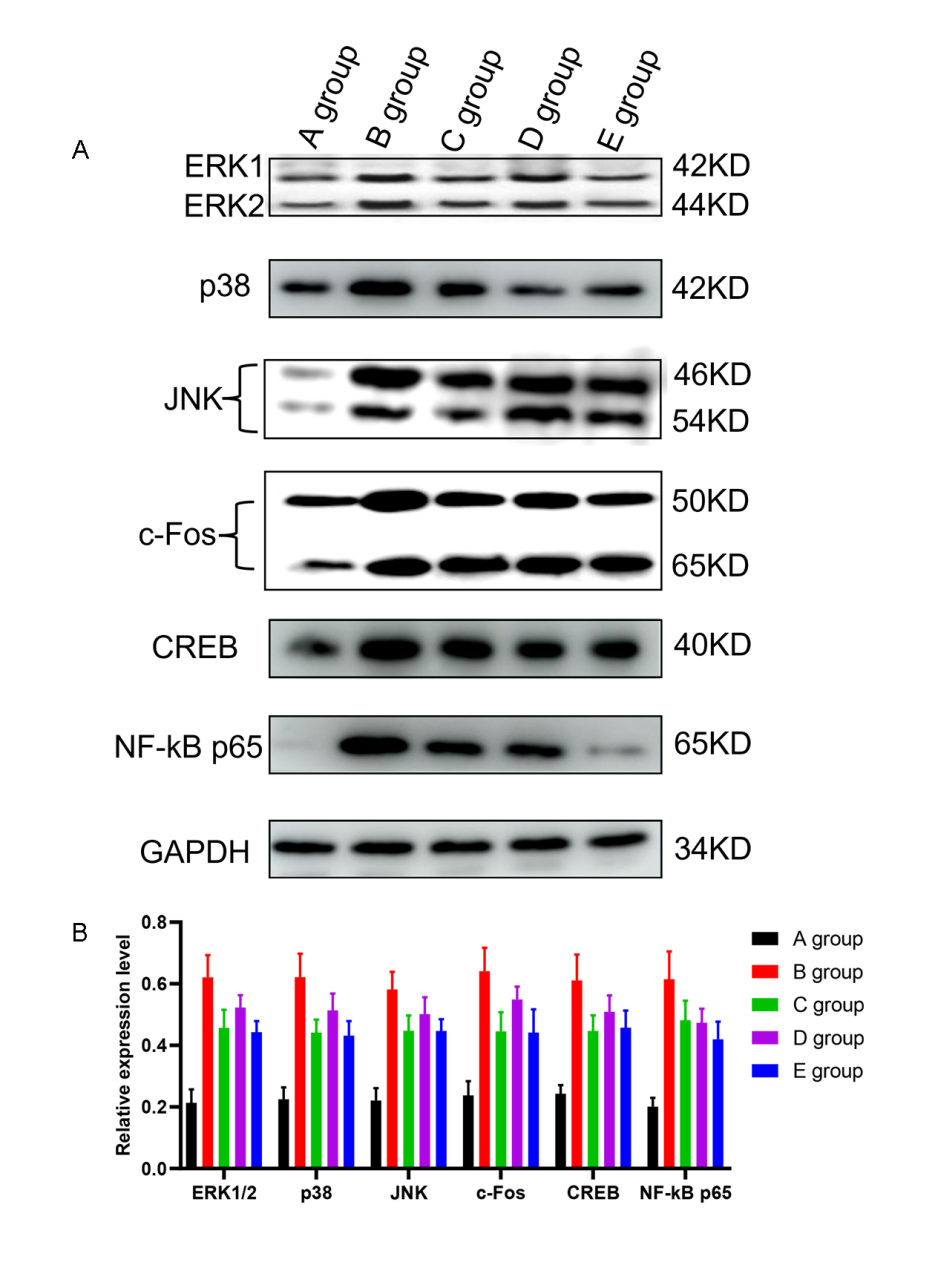
**The effect of Huangqi Guizhi Wuwu Decoction on the expression of ERK1/2, p38, JNK, c-Fos, CREB and NF-kB in the L4-L5 dorsal root ganglions of different rat groups detected by western blotting.** (**A**) The expression of ERK1/2, p38, JNK, c-Fos, CREB, NF-kB and GAPDH in different rat groups. (**B**) The relative expression levels of ERK1/2, p38, JNK, c-Fos, CREB, and NF-kB compared to GAPDH in different rat groups.

Compared with group A, the expression of ERK1/2, p38, JNK, c-Fos, CREB, and NF-kB in the L4-L5 dorsal root ganglions in group B was significantly higher (*P*<0.001, Figure 7). Compared with group B, the expression of ERK1/2, p38, JNK, c-Fos, CREB, and NF-kB in the L4-L5 dorsal root ganglions in groups C-E was also significantly lower (*P*=0.021, *P*=0.018, *P*=0.019, *P*=0.020, *P*=0.017, *P*=0.018; *P*=0.025, *P*=0.029, *P*=0.030, *P*=0.028, *P*=0.027, *P*=0.019; *P*=0.021, *P*=0.018, *P*=0.019, *P*=0.021, *P*=0.019, *P*=0.015, respectively, Figure 7). And group C and group E exhibited the most melancholic expression of ERK1/2, p38, and c-Fos.

**Figure 8:**
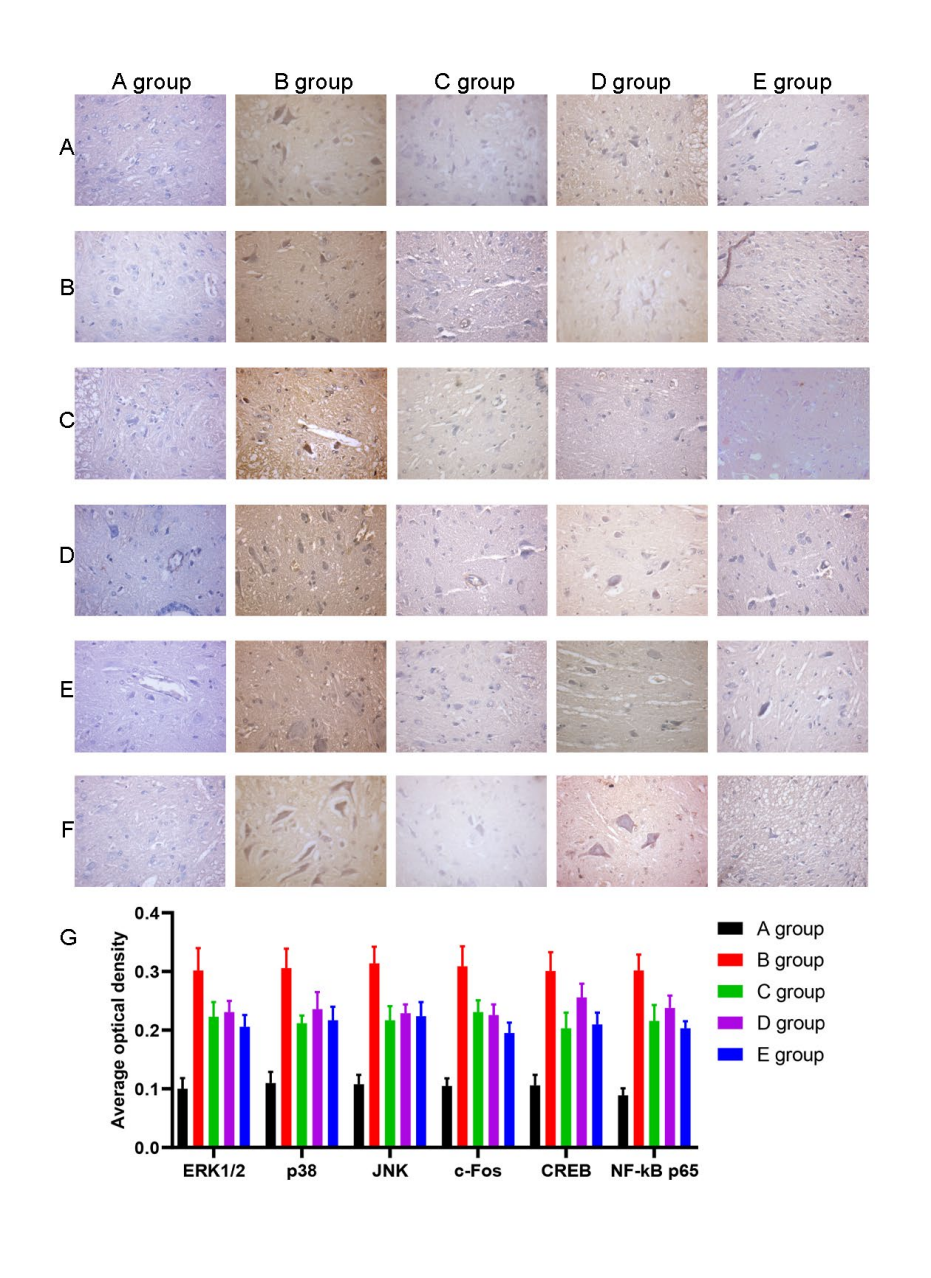
**The effect of Huangqi Guizhi Wuwu Decoction on the expression of ERK1/2, p38, JNK, c-Fos, CREB and NF-kB in the L4-L5 dorsal root ganglions of different rat groups detected by IHC.** (**A**–**F**) The typical pictures of ERK1/2, p38, JNK, c-Fos, CREB and NF-kB expression in the L4-L5 dorsal root ganglions of different rat groups detected by IHC. (**G**) The average optical density of ERK1/2, p38, JNK, c-Fos, CREB, and NF-kB expression in the L4-L5 dorsal root ganglions of different rat groups.

We also examined the expression of ERK1/2, p38, JNK, c-Fos, CREB, and NF-kB by IHC. The average optical density of ERK1/2, p38, JNK, c-Fos, CREB, and NF-kB expression was higher in group B than in group A (*P*<0.001). The average optical density of ERK1/2, p38, JNK, c-Fos, CREB, and NF-kB expression was lower in groups C-E than group B (*P*=0.020, *P*=0.017, *P*=0.018, *P*=0.019, *P*=0.016, *P*=0.017; *P*=0.026, *P*=0.028, *P*=0.029, *P*=0.027, *P*=0.026, *P*=0.018; *P*=0.020, *P*=0.017, *P*=0.018, *P*=0.020, *P*=0.018, *P*=0.014, respectively, Figure 8).

These results suggest that by lowering the levels of TNFα, IL-1β, and IL-6 in the serum of oxaliplatin CINP model rats, Huangqi Guizhi Wuwu Decoction suppresses the expression of MAPK signaling pathway related proteins such as ERK1/2, p38, and JNK. It also down-regulates the expression of downstream essential proteins, including c-Fos, CREB and NF-kB, combating nerve cell injury, reducing pain sensitization, and preventing and repairing the harm of oxaliplatin in nerve cells.

## DISCUSSION

The target of oxaliplatin chronic neurotoxicity is spinal dorsal root ganglion. After entering the body, the drug first accumulates in the dorsal root ganglion of the spinal cord. Then, it produces oxidative stress and mitochondrial damage, which can result in the apoptosis of nerve cells. Subsequently, neurological dysfunction and neuropathic pain can occur [22–24]. The present study found that Huangqi Guizhi Wuwu Decoction can improve the slow weight gain of oxaliplatin-induced chronic CINP model rats, effectively preventing and treating the oxaliplatin-induced regular CIPN rat model of hyperalgesia. It can also oppress the mechanical pain threshold, cold pain threshold, and heat pain threshold decreased. The effect of Huangqi Guizhi Wuwu Decoction external immersing was better than that of Huangqi Guizhi Wuwu Decoction gavage. Huangqi Guizhi Wuwu Decoction could effectively antagonize the effect of oxaliplatin on the injury of spinal dorsal root ganglion cells in rats.

Previous studies have found that TNFα, IL-1β, and IL-6 played a role in neuropathic pain and could decrease the excitability of neurons via MAPK/p38 pathway to alleviate mechanical hyperalgesia in rats [25–27]. MAPK/NF-κB was reported to be a critical signaling pathway involved in regulating inflammation-related neural pathogenesis [28, 29]. Li Tao and his colleagues also found that MAPKs, ERK, JNK, and p38, were involved in the inflammation responses after acute brain injury [30]. It was also suggested that NF-κB activation could contribute to pro-inflammation cytokines, and these cytokines can, in turn, activate NF-κB [31]. This positive feedback loop was believed to amplify inflammatory signals. As for the relationships between MPAK and NF-kB, MAPK was considered to be a classical pathway that could initiate NF-kB activation [32]. The activation of MAPK-NF-kB was reported to promote TNFα, IL-1β, and IL-6 [33]. This also formed a positive feedback loop. These findings suggest that TNFα/IL-1β/IL-6/MAPK/NF-kB plays a vital role in the development and maintenance of neuropathic pain. In this study, we found that Huangqi Guizhi Wuwu Decoction down-regulates the levels of TNF-α, IL-1β, IL-6 in the serum of chronic CINP rats induced by oxaliplatin, then suppresses the expression of MAPK pathway related-proteins such as ERK1/2, p38, and JNK. Subsequently, the expression of downstream essential proteins, including c-Fos, CREB, and Nf-κB also decreases. Consequently, Huangqi Guizhi Wuwu Decoction combats nerve cell injury, reduces pain sensitization, and prevents and repairs nerve cell injury in oxaliplatin CINP model rats.

It is important to note that this study had some limitations. We only analyzed the change of MAPK signal pathway in the treatment process of Huangqi Guizhi Wuwu Decoction on CINP. It would be logical to speculate that there might be other essential pathways that can regulate this process. Therefore, we plan to investigate other potential critical pathways by using the high throughput sequencing method in the future. We hope our research results can contribute to the development and application of traditional Chinese medicine.

In conclusion, we found that Huangqi Guizhi Wuwu Decoction can combat nerve cell injury, reduce pain sensitization, and prevent and repair the damage of nerve cells in oxaliplatin CINP model rats via TNFα/IL-1β/IL-6/MAPK/NF-kB pathway. These findings suggest that Huangqi Guizhi Wuwu Decoction may be used in clinical practice to effectively treat oxaliplatin CINP.
